# Clinical Insights Into Lower Incisor Extraction for Orthodontic Treatment: A Case Report

**DOI:** 10.7759/cureus.69877

**Published:** 2024-09-21

**Authors:** Mrudula Shinde, Pallavi Daigavane, Ranjit Kamble, Nikhil Kumar, Nishu Agarwal, Dhwani Suchak, Utkarsha Chaudhari, Aathira Surendran, Prerana A Suryawanshi

**Affiliations:** 1 Orthodontics and Dentofacial Orthopaedics, Sharad Pawar Dental College and Hospital, Datta Meghe Institute of Higher Education and Research, Wardha, IND; 2 Orthodontics and Dentofacial Orthopaedics, Kusum Devi Sunderlal Dugar Jain Dental College and Hospital, Kolkata, IND; 3 Orthodontics and Dentofacial Orthopaedics, Nair Hospital Dental College, Mumbai, IND

**Keywords:** class i malocclusion, lower incisor extraction, molar uprighting, overbite, overjet

## Abstract

Extracting a single lower front tooth can be an appropriate treatment for class I malocclusions when the alignment of the upper teeth is normal and there is an adequate overlap of the teeth. This approach is particularly effective in cases of significant crowding in the lower front teeth, especially when the space deficiency exceeds 4-5 mm and the combined width of the lower front teeth surpasses 83 mm. It is also a viable option for malocclusions resulting from discrepancies in tooth size, such as narrower upper front teeth or larger lower front teeth. Research suggests that this method leads to better post-treatment stability compared to the conventional approach of premolar extraction. The success of this treatment is contingent upon meticulous diagnosis, comprehensive planning, and the expertise of the orthodontic professional. This method not only addresses specific issues of space and alignment but also provides a more stable and predictable long-term outcome for patients with these particular orthodontic challenges.

## Introduction

The integration of extractions into orthodontic treatment is a well-established concept within the field. In order to achieve a harmonious functional occlusion that complements the contiguous musculature and supporting structures, the extraction of one or more teeth may be necessary. The decision to proceed with extractions is determined by specific clinical criteria, including variations between the dental and basal arches, the facial profile, and the general condition of the dentition in relation to the cranial base. Given the inherent variability in defining what constitutes a normal occlusion, treatment approaches must be individualized to address each patient's unique requirements.

Hopkins noted that crowding of mandibular incisors is a common occurrence during normal growth. In a comprehensive study of 300 malocclusions [1], Neff discovered that the upper anterior teeth are 18-36% larger than the lower anterior teeth. Consequently, he recommended compensatory measures for segments that lack harmony. Furthermore, he suggested that the successful treatment of three-incisor cases offers valuable insights that could be consciously utilized in addressing specific malocclusions [2]. Many of the challenges we encounter necessitate compromise solutions, as achieving perfection is often precluded by inherent biological variations among patients.

Orthodontic treatment can still achieve optimal denture design, provided that the resulting product is both functionally and aesthetically harmonious and stable for the specific circumstances. We posit that a stable, harmonious orthodontic outcome hinges on a sound occlusion, which not only sustains the individual components but also fosters a healthy periodontium, temporomandibular joint, and neuromuscular mechanism [3].

The extraction of lower incisors has conventionally been performed in cases where the incisors are ectopically positioned or have a poor prognosis [4]. However, given the current range of treatment alternatives, the carefully selected extraction of a single incisor can contribute to achieving optimal outcomes through the application of fundamental treatment mechanics [5].

This surgical procedure is most suitable for individuals presenting with class I dental malocclusions, characterized by significant lower anterior crowding. Ideal candidates possess a normal maxillary dentition, significant lower anterior crowding (insufficient space for approximately one lower incisor), and optimal buccal interdigitation. Furthermore, it is advised for class I individuals with anterior dental cross-bite originating from lower anterior crowding and incisor protrusion, as well as those exhibiting acute anterior tooth-size discrepancies due to small upper or large lower incisors [2].

Conversely, this procedure is not recommended for cases featuring deep bites and horizontal growth patterns, upper first premolar extraction while canines are in a class I relationship, bimaxillary crowding devoid of incisor tooth-size mismatches, or incisor discrepancies resulting from small lower incisors or large maxillary incisors.

Which incisor?

In the determination of which lower incisor to extract, it is essential to consider various variables, including the extent of anterior arch length deficiency, the ratio of anterior teeth, periodontal and tooth health, and the alignment of the upper and lower midlines.

## Case presentation

A 16-year-old female patient presented at the Department of Orthodontics and Dentofacial Orthopedics at Sharad Pawar Dental College and Hospital, Wardha, India, with a significant concern regarding the malposition of her mandibular teeth, as documented during the orthodontic clinic assessment. A comprehensive habit history and clinical examination were conducted, and the patient's pre-treatment records, including radiographs, study models, and photographs, were obtained.

**Figure 1:**
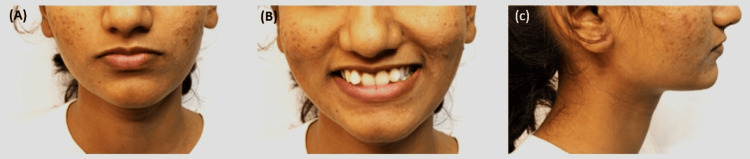
Pretreatment extraoral photograph: (A) frontal, (B) smiling, (C) left profile

Upon clinical examination, the patient's extraoral assessment revealed a symmetrical mesoprosopic face form with well-positioned lips. Notably, the upper lip exhibited hypotonicity, while the lower lip displayed eversion. The patient presented with a straight facial profile, and her smile demonstrated symmetrical and harmonious characteristics, along with a full incisor display (Figure 1).

**Figure 2:**
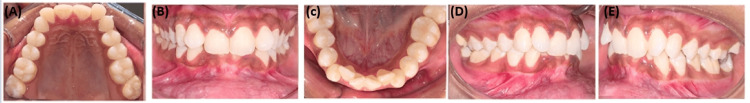
Pretreament intraoral photographs: (A) maxillary arch, (B) anteriors in occlusion, (C) mandibular arch, (D) right lateral in occlusion, (E) left lateral in occlusion

Upon conducting an intraoral examination, it was observed that all teeth up to the second molar were present in both dental arches. Gingival health was deemed satisfactory, and an adequate zone of connected gingival tissue was present. Except for the third molars, all teeth were present in both arches. Notably, the mandibular second molar on the left side exhibited mesial inclination. The molars and canines exhibited a class I relationship. Reduced overjet and increased overbite were noted. Functional evaluation revealed normal speech, oro-nasal breathing, and a typical swallowing pattern. Mandibular closure proceeded without deviation, and no temporomandibular disorder (TMD)-related signs or symptoms were observed (Figure 2).

**Figure 3:**
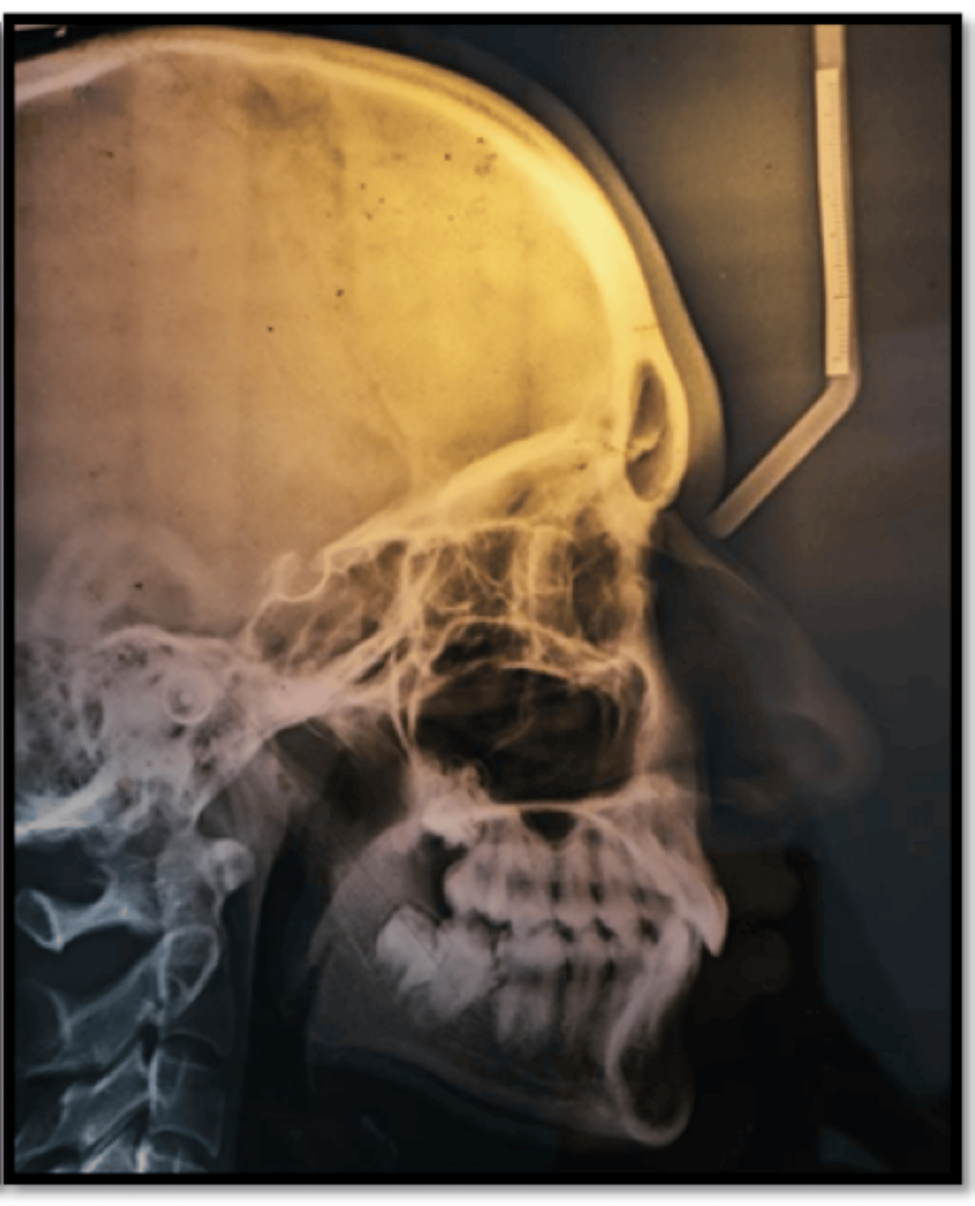
Pretreatment radiograph: lateral cephalogram

Cephalometric analysis revealed that the patient was determined to be in cervical vertebral maturation index stage VI (maturation) and exhibited class I skeletal bases, indicating horizontal growth. In addition, it was observed that the upper incisors displayed retroclination. Furthermore, the soft tissue investigation revealed an average nasolabial angle and a deep mentolabial sulcus (Figure 3).

The principal objective of the procedure was to address the retroclination of the upper incisors, alleviate lower arch crowding, and reposition the mandibular left second molar. In addition, the aim was to establish a proper occlusion, close any existing interdental spaces, and achieve optimal overjet and overbite. The ultimate goal was to realize harmonious facial aesthetics and balance, along with the cultivation of an aesthetically pleasing smile. Individuals diagnosed with class I dental malocclusions, particularly those exhibiting significant crowding in the lower anterior region, have a normal maxillary dentition and optimal buccal interdigitation and demonstrate marked discrepancies in mandibular anterior tooth size, suggesting single tooth extraction in the lower anterior region.

**Figure 4:**
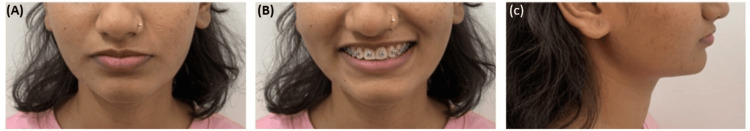
Stage extraoral photograph: (A) frontal, (B) smiling, (C) left lateral profile

**Figure 5:**
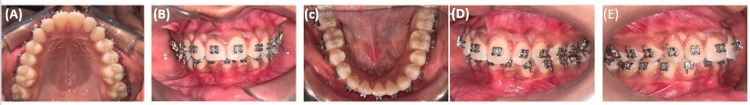
After the retraction phase: (A) maxillary arch, (B) anteriors in occlusion, (C) mandibular arch, (D) left lateral in occlusion, (E) right lateral in occlusion

The proposed treatment plan involves the utilization of fixed mechanotherapy. To address the critical anchorage issue in both the upper and lower arches, treatment commenced using a pre-adjusted edgewise appliance (MBT 0.022" bracket slot, supplied by Liberal Traders, New Delhi). The mandibular right lateral incisor was extracted from its lingual position. Alignment was initiated with a 0.016" nickel-titanium wire in both the maxillary and mandibular arches, advancing to a 0.017 × 0.025'' stainless steel wire over a four-month alignment period for closure of extraction space (Figure 4 and Figure 5).

**Figure 6:**
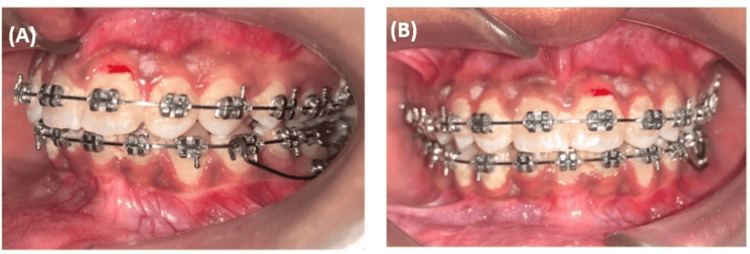
Mandibular left second molar correction with uprighting spring: (A) left lateral occlusion, (B) anterior occlusion

**Figure 7:**
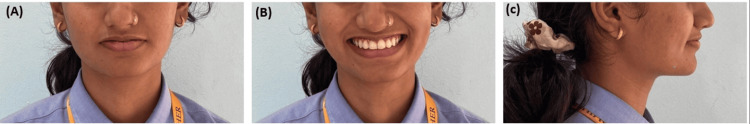
Post-treatment extraoral photograph: (A) frontal, (B) smiling, (C) left profile

**Figure 8:**
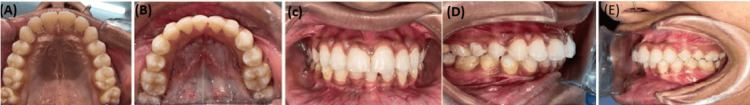
Postoperative intraoral photographs: (A) maxillary arch, (B) mandibular arch, (C) anterior in occlusion, (D) right lateral occlusion, (E) left lateral occlusion

The mesially inclined mandibular left second molar was successfully corrected using an uprighting spring, as illustrated in (Figure 6). Following the completion of the treatment, the patient's primary concerns regarding irregularly positioned lower teeth and the retroclined upper anterior teeth were effectively addressed. The result is a balanced smile, satisfactory lip positioning, and the preservation of class I molar relationships on both sides, as depicted in Figure 7 and Figure 8.

**Figure 9:**
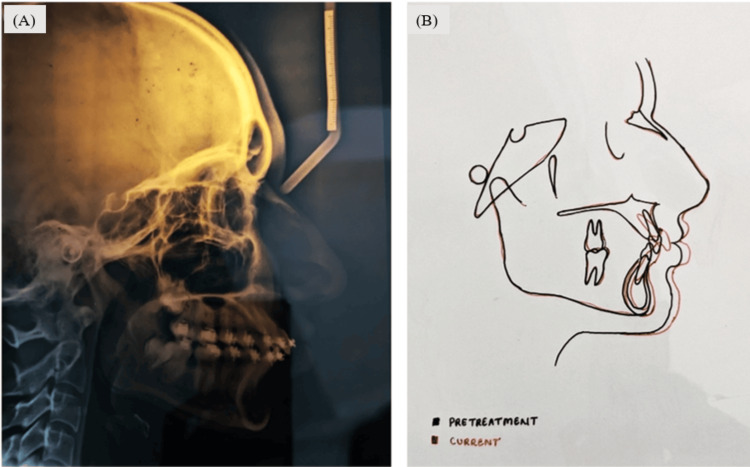
Post-treatment radiographic evaluation: (A) postoperative cephalogram, (B) superimposition of pretreatment and postoperative cephalogram

Optimal overjet and overbite, as well as enhanced facial aesthetics and balance, were achieved. Both pre- and post-treatment photographic documentation and radiographic imagery demonstrate a significant improvement in profile, overjet, and overbite, as shown in Figure 9.

Furthermore, In cases of single mandibular incisor extraction during orthodontic treatment, other options could be considered based on the tooth-arch discrepancy. If the discrepancy is minimal, interproximal reduction (IPR) or arch expansion could create sufficient space for alignment. In cases of moderate discrepancy, a proclination of the lower incisors might be effective. Alternatively, space maintainers or orthodontic camouflage can be employed to manage space without extraction. For more precise control, lingual braces or clear aligners can be used to gradually align the teeth. The choice of treatment should be tailored to the specific needs and goals of the patient. the procedure contributed to improved smile aesthetics, correction of retroclination in the upper teeth, and alleviation of crowding in the lower anterior teeth.

## Discussion

Extractions have been a longstanding method in orthodontics for creating space. In 1905, Jackson pioneered the recommendation for lower incisor excision to alleviate mandibular crowding [6]. The strategic removal of lower incisors is particularly advantageous in cases of extreme crowding, as it enhances stability in the anterior region. However, it is essential to exercise careful and precise case selection in instances where six maxillary upper teeth and five mandibular lower teeth occlude, especially in the presence of Bolton's anterior tooth material surplus in the mandibular arch and a deep bite [7]. Moreover, in cases where further growth potential is limited, the consideration of extraction plays a pivotal role in achieving optimal treatment outcomes.

Riedel proposed that the removal of one or more mandibular incisors may offer added stability to the mandibular anterior dental arch in situations where permanent retention is not feasible [7]. Salzman postulated that extracting a mandibular incisor could potentially result in a significant overbite [8]. Kokich and Shapiro underscore the importance of a comprehensive review of all diagnostic records before considering this treatment strategy to mitigate the risk of exacerbating overbite. Specifically, patients presenting with mandibular anterior tooth size excess (referred to as Bolton's disharmony) should undergo evaluation for possible mandibular incisor extraction. In addition, reproximation of the maxillary anterior teeth may be necessary to establish the correct overbite and overjet [9].

In the realm of orthodontics, a longstanding debate pertains to the decision of whether to extract teeth as part of the treatment process. While addressing malocclusions through asymmetric extractions is atypical, there are situations in which treatment objectives may need to be modified based on the individual patient's needs, even if the final occlusal alignment may be suboptimal. Commonly, extraction of first or second premolars is undertaken to resolve tooth-size arch-length disparities or crowding. An alternative approach involves the extraction of first or second molars. Incisor extractions may also be carried out to address mandibular anterior crowding [10].

Valinoti has observed that the extraction of a lower incisor is less likely to result in relapse following retention due to several key factors. First, the proximity of the incisor to the area of crowding necessitates minimal tooth movement, thereby preserving larger areas of the teeth's original positions. Second, there is a reduced load on the anchor teeth during the space closure process, as the majority of the space is utilized for anterior correction. Third, muscle pressure does not induce instability, as there is minimal interaction between the tongue and lips with the unaltered tooth position. In addition, the maintenance or only minor alteration of intercanine width has been noted. These findings suggest that lower incisor extraction offers a stable and effective solution for addressing dental crowding, with fewer complications related to post-treatment relapse [3].

## Conclusions

The strategic extraction of a single mandibular incisor can serve as a viable therapeutic modality for specific malocclusions, aiming to establish both functionally and aesthetically harmonious dentition. This approach is most applicable to individuals presenting class I malocclusions, normal maxillary dentition, robust buccal interdigitation, and substantial lower anterior crowding, particularly when confronted with a lower anterior arch length deficiency exceeding 4-5 mm and an anterior tooth ratio surpassing 83 mm. When appropriately recommended and executed, the extraction of a lower incisor can markedly ameliorate select malocclusions and yield superior orthodontic outcomes, ensuring functional occlusal harmony.

## References

[1] Hopkins S (1955). Inadequacy of mandibular anchorage. Am J Orthod.

[2] Neff C (1957). The size relationship between the maxillary and mandibular anterior segments of the dental arch. Angle Orthod.

[3] Valinoti J (1994). Mandibular incisor extraction therapy. Am J Orthod Dentofacial Orthop.

[4] Klein D (1997). The mandibular central incisor, an extraction option. Am J Orthod Dentofacial Orthop.

[5] Bayram M, Özer M (2007). Mandibular incisor extraction treatment of a class I malocclusion with Bolton discrepancy: a case report. Eur J Dent.

[6] Jackson V (1904). Orthodontia and orthopaedia of the face. Lippincott 1904.

[7] Riedel RA, Little RM, Bui TD (1992). Mandibular incisor extraction--postretention evaluation of stability and relapse. Angle Orthod.

[8] Salzmann J (1949). Criteria for extraction in orthodontic therapy related to dentofacial development. Am J Orthod.

[9] Kokich VG, Shapiro PA (1984). Lower incisor extraction in orthodontic treatment. Four clinical reports. Angle Orthod.

[10] Garg H, Khatria H, Kaldhari K, Singh K, Purwar P, Rukshana R (2021). Intermolar and intercanine width changes among Class I and Class ii malocclusions following orthodontic treatment. Int J Clin Pediatr Dent.

